# Skin cancer screening participation and impact on melanoma incidence in Germany – an observational study on incidence trends in regions with and without population-based screening

**DOI:** 10.1038/bjc.2012.22

**Published:** 2012-01-31

**Authors:** A Waldmann, S Nolte, M A Weinstock, E W Breitbart, N Eisemann, A C Geller, R Greinert, B Volkmer, A Katalinic

**Affiliations:** 1 University Hospital Schleswig-Holstein, Campus Luebeck, Institute of Clinical Epidemiology, Ratzeburger Allee 160, 23562 Luebeck, Germany; 2 Association of Dermatological Prevention (ADP) e. V., Cremon 11, 20457 Hamburg, Germany; 3 Deakin University, Burwood, Victoria, Australia; 4 Dermatoepidemiology Unit, V A Medical Center Providence; Department of Dermatology, Rhode Island Hospital, and Departments of Dermatology and Epidemiology, Brown University, Providence, RI, USA; 5 Elbe Clinics, Center of Dermatology, Am Krankenhaus 1, 21614 Buxtehude, Germany; 6 University of Luebeck, Institute of Cancer Epidemiology, Ratzeburger Allee 160, 23562 Luebeck, Germany; 7 Department of Society, Human Development, and Health, Harvard School of Public Health, Boston, MA, USA

**Keywords:** skin neoplasms, mass screening, incidence, melanoma, epidemiologic studies

## Abstract

**Background:**

The SCREEN (Skin Cancer Research to provide Evidence for Effectiveness of Screening in Northern Germany) project involved population-wide skin cancer screening with whole-body examination by general physicians and dermatologists. It was conducted in the German state of Schleswig-Holstein (July 2003–June 2004), but not in the German state of Saarland.

**Methods:**

The population-based registries of Schleswig-Holstein and Saarland provided data on melanoma incidence before, during, and after SCREEN to assess the association of skin cancer screening with incidence.

**Results:**

Approximately 19% of the Schleswig-Holstein population participated in SCREEN (women: 27%, men: 10%). A total of 52% of all melanomas diagnosed during SCREEN in Schleswig-Holstein were detected as part of the project. Melanoma incidence increased during SCREEN (invasive melanoma in women: +8.9 per 100 000 (95% confidence intervals (CI): 6.1; 11.7); men: +4.0 per 100 000 (95% CI: 1.6; 6.4)) and decreased afterwards (women: −10.6 per 100 000 (95% CI: −13.3; −7.9); men: −4.1 per 100 000 (95% CI: −6.5; −1.7)). Similar changes were not observed in Saarland that had no such project. The differences between the two states were greatest among women, the group with the greater SCREEN participation.

**Conclusion:**

The SCREEN project had a substantial impact on melanoma incidence. This is consistent with the impact of effective screening for other cancers.

Cutaneous melanoma is a public health problem of increasing magnitude among light-skinned populations worldwide (Geller *et al*, 2007; Garbe and Leiter, 2009; Horner *et al*, 2009; RKI and GEKID, 2010). In 2003–2004, the SCREEN project (Skin Cancer Research to provide Evidence for Effectiveness of Screening in Northern Germany) was conducted in Schleswig-Holstein, Germany. The success of this feasibility project (Breitbart *et al*, 2011) led to the implementation of the national Skin Cancer Early Detection Program. This programme has an initial 5-year trial period and commenced in July 2008.

In population-based screening programmes such as those for breast or cervical cancer, incidence increases after the uptake of screening, due to the detection of prevalent cancers and other factors (Canfell *et al*, 2006; Anttila *et al*, 2008; Hofvind *et al*, 2008; van der Aa *et al*, 2008; Pollan *et al*, 2009, 2010), and declines when screening activities end.

To date, there are no reports on incidence effects of population-based skin cancer screening programmes using whole-body examination. Herein, we document participation in the SCREEN project and population-based melanoma incidence rates before, during, and after SCREEN in Schleswig-Holstein. Incidence is compared with Saarland, a federal state in southwestern Germany with a long tradition of and a high-completeness of cancer registration, but without a population-based screening during the SCREEN's inception in 2003.

## Materials and methods

### The SCREEN project

The population-based SCREEN project was a 12-month skin cancer screening intervention (whole-body examination conducted by either general physicians and/or dermatologists; July 2003–June 2004). Eligible persons were residents of Schleswig-Holstein, aged ⩾20 years, and policyholders of statutory health insurance. Persons receiving skin cancer care were not eligible. Participants chose between non-dermatologist physicians and dermatologists for their initial whole-body skin examination. If a non-dermatologist found a suspicious skin lesion or classified the screenee as at increased risk for skin cancer, he was then referred to a dermatologist to receive a second whole-body examination. Only dermatologists were allowed to excise lesions. All screening physicians received a mandatory 8-h training course before the SCREEN (Breitbart *et al*, 2011).

Ethics approval was not required as SCREEN was an integral part of the standard medical care. The SCREEN participants gave written informed consent for data storage and analysis; participation was voluntary.

### Statistical analysis

Crude tumour detection rates are given as melanoma cases per 100 000 screenees. Melanomas reported to the Cancer Registry are presented as age-standardised incidence rates per 100 000 inhabitants (ASR Europe). Poisson-based confidence intervals (95% CI) were computed according to Dobson *et al* (1991). Student's *t*-tests (R) provided approximate *P*-values for the comparison of absolute changes in Schleswig-Holstein and Saarland (R Development Core Team, 2010).

Several time periods were defined for the analysis: pre-SCREEN (January 1998–December 2000; period without screening in Schleswig-Holstein), SCREEN (July 2003–June 2004; period in which screening was conducted in Schleswig-Holstein), and post-SCREEN (January 2005–December 2007; period without formal screening activities). The years 2001–2002 were not included, because pilot projects in advance of the full-scale SCREEN project began in Schleswig-Holstein in 2001.

## Results

### Participation in SCREEN

Of 1.88 million eligible citizens, 19.2% received skin cancer screening during the 1-year screening period (27.0% of women, 10.4% of men; Table 1).

### Melanoma findings in Schleswig-Holstein

During the SCREEN period, 1116 incident melanomas (31% *in situ*) were reported to the Schleswig-Holstein Cancer Registry (Table 2). Of these, 585 melanomas (also 31% *in situ*) were detected as part of the SCREEN project, equaling 52% of all melanomas reported during this period (56% in women, 46% in men).

Although men comprised 26.4% of all screenees, they represented 36.1% of all 585 melanomas detected during the SCREEN. The difference between the proportion of all 360 288 screenees and the proportion of all SCREEN-detected melanomas was highest in the subgroup of men aged ⩾50 years (16% of all screenees, 29% of all SCREEN-detected melanomas; Table 1).

### Time trends in melanoma incidence rates

Throughout all time periods, the incidence of *in situ* and invasive melanomas was higher in Schleswig-Holstein than in Saarland (Table 3). Women in Schleswig-Holstein compared with those from Saarland experienced sharper increases in incidence during the SCREEN period and sharper decreases in incidence in the post-SCREEN period. In Schleswig-Holstein women, the incidence changes (e.g., absolute differences in incidence rates) were substantial and statistically significant. In Schleswig-Holstein men, incidence changes were significant and similar in direction, but less pronounced compared with women. The differences between Schleswig-Holstein and Saarland men regarding the absolute differences in incidence rates were neither in the SCREEN period nor in the post-SCREEN period significant.

## Discussion

The SCREEN project was a population-based skin cancer-screening programme using whole-body examinations. Although incidence changes were observed in Schleswig-Holstein in a temporal association with SCREEN activities, no such changes were seen in Saarland (region without SCREEN; exception in men changes *in-situ* melanoma incidence). Differences between the two regions were greatest among women, the group with higher participation in SCREEN.

### Participation in SCREEN

We sought to compare participation rates from Schleswig-Holstein with earlier surveys that ascertained whether they ‘ever had skin cancer screening’ or not. The proportion of people reporting to have had skin cancer screening in both Austria and the US was somewhat lower than the participation proportion achieved in the 1-year SCREEN project (Haidinger *et al*, 2009; Lakhani *et al*, 2009). As known from other cancer early-detection programmes, some population groups (e.g., women, high socio-economic status, and urban dwellers) are more responsive to such programmes than others (Scheffer *et al*, 2006; von Wagner *et al*, 2009; Palencia *et al*, 2010; Sprague *et al*, 2010). Likewise in SCREEN, women show a substantially higher participation compared with men (Haidinger *et al*, 2009). Similar patterns have been observed in the American Academy of Dermatology National Melanoma/Skin Cancer Screening Program (Goldberg *et al*, 2007) or the German colonoscopy screening (Altenhofen *et al*, 2009).

Men who participate in skin cancer screening programmes tend to be high-risk individuals (Jemal *et al*, 2007; Paoli *et al*, 2009; Breitbart *et al*, 2011). In the American Academy of Dermatology programme, men ⩾50 years comprised 23% of all screenees, but accounted for 32% of all suspected melanomas (Goldberg *et al*, 2007).

### Melanoma epidemiology

A significant increase in melanoma incidence was observed between pre SCREEN and SCREEN, whereas in the 3-year period following SCREEN, especially invasive melanoma incidence returned to baseline levels. In contrast in Saarland, we observed a general small incidence increase from 2000 to 2007 for both *in situ* and invasive melanomas.

The significant increase of *in situ* melanomas in men reported here can be contributed to a chance finding due to small absolute numbers of *in situ* melanomas and year-to-year fluctuation. In Saarland, *in situ* incidence has been increasing steadily since 1980. However, from 1998 to 2002, a ‘dent’ is seen in the *in situ* incidence, generating the significant effect. In sensitivity analyses with differing time periods as Saarland's baseline incidence, the increase was non-significant.

The observed increase in incidence in Schleswig-Holstein is consistent with the hypothesis that after implementing a screening programme, an incidence rise (Hofvind *et al*, 2008), and with findings from breast cancer and cervical cancer screening interventions (Anttila *et al*, 2008; Hofvind *et al*, 2008; van der Aa *et al*, 2008; Pollan *et al*, 2010). The absolute change in incidence during the SCREEN period was higher in women than in men, as were respective proportions of the population participating in SCREEN. However, men with clinical symptoms are more likely to attend to skin cancer screening (Geller *et al*, 2002; Janda *et al*, 2006; Breitbart *et al*, 2011). This may have contributed to the increase in invasive melanoma incidence, but not in melanoma *in situ* incidence on the population level.

The incidence differences observed within Germany with the highest incidence rates in northern Germany (e.g., Schleswig-Holstein) and lower rates in the south (e.g., Saarland) match the general European pattern with the highest incidence rates in Scandinavia and lower incidence rates in Central Europe (WHO and IARC, 2011).

### Strengths and limitations

An important strength of the SCREEN project is the high acceptance among both the population and the physicians (98% of dermatologists and 64% of eligible non-dermatologists participated (Breitbart *et al*, 2011)). Further, the population-based Cancer Registries of Schleswig-Holstein and Saarland have an excellent history of cancer registration, with both registering more than 95% of all expected melanomas. SCREEN offers the opportunity to compare incidence in a region with and a region without population-based skin cancer screening pre-, during, and post-screening activities.

As not all statutory health insurances participated in SCREEN, 1.65 million of 1.88 million adults living in Schleswig-Holstein were eligible to be screened. However, the true age- and sex-distribution is only known for the total population of 1.88 million adults. Hence, the estimate of a 19% participation rate (i.e., persons with a documented SCREEN examination in the total population of 1.88 million) might be too conservative. Furthermore, patients without informed consent, policyholders of non-participating health insurances, or patients who saw their physician for other health reasons might also have benefitted from the improved ability of SCREEN physicians to detect skin cancer.

Further, we assume that an *ad-hoc* participation rate of at least 19% in the first year and the 50% capture rate of all melanomas reported to the Cancer Registry Schleswig-Holstein within SCREEN could impact the incidence as reported herein.

## Conclusion

The SCREEN programme was associated with increased melanoma incidence compared with the pre- and post-SCREEN rates in Schleswig-Holstein and compared with Saarland, where population-based screening did not take place. This effect was strongest among women who were nearly three times as likely to participate as men. The reported experience is consistent with observations in effective breast and cervical cancer screening (Canfell *et al*, 2006; Anttila *et al*, 2008; Hofvind *et al*, 2008; van der Aa *et al*, 2008; Pollan *et al*, 2009, 2010). It suggests that SCREEN was a similarly effective programme for melanoma.

## Figures and Tables

**Table 1:** Description of SCREEN project participants, confirmed diagnoses of MM (absolute, relative frequencies), and population-based participation rates

	**Women (*n*=265 306)**	**Men (*n*=94 982)**
Mean age (s.d.)	48.2 (16.2)	53.9 (15.7)
		
*Age group (years) (n (%))* a		
⩽34	59 513 (22.4)	12 043 (12.7)
35–49	86 375 (32.6)	25 133 (26.5)
50–64	70 117 (26.4)	29 765 (31.3)
⩾65	49 301 (18.6)	28 050 (29.5)
		
*Number of histopathologically confirmed MM (*n *(%))*^a^		
In total	372 (0.14)	213 (0.22)
Stratified according to age-group (years)		
⩽34	58 (0.09)	18 (0.15)
35–49	118 (0.13)	24 (0.09)
50–64	109 (0.16)	72 (0.24)
⩾65	87 (0.18)	99 (0.35)
		
*Crude MM detection rates per 100 000 screenees* b		
In total	140.2	221.1
Stratified according to age-group (years)		
⩽34	97.5	149.5
35–49	136.6	91.5
50–64	155.5	241.9
⩾65	176.5	345.8
		
*Population-based participation (%)* c		
In total	27.0	10.4
Stratified according to age-group (years)		
⩽34	29.0	5.7
35–49	30.6	8.6
50–64	30.0	12.8
⩾65	19.0	15.5

Abbreviations: MM=melanoma; SCREEN=Skin Cancer Research to provide Evidence for Effectiveness of Screening in Northern Germany.

a Percentages are based on the total number of screenees in sex and sex-by-age-groups, respectively.

b Number of MM findings in a particular subgroup/divided by number of screenees in subgroup multiplied by 100 000.

c Percentages are based on the total number of eligible persons in Schleswig-Holstein in sex and sex-by-age groups, respectively.

**Table 2:** MM that were reported to the cancer registry Schleswig-Holstein for the SCREEN period and the proportion of melanomas detected during SCREEN

	**Women**	**Men**
*MM reported to cancer registry for the SCREEN period*		
Total number of MM	659	457
*In situ* MM (*n* (%))	220 (33.4)	127 (27.8)
Invasive MM (*n* (%))	439 (66.6)	330 (72.2)
		
*Histopathologically confirmed MM in SCREEN*		
Total number of MM	372	210
*In situ* MM (*n* (%))	131 (35.2)	49 (23.3)
Invasive MM (*n* (%))	241 (64.8)	161 (76.7)
		
*Proportion of MM that were detected during SCREEN (%)*		
All MM	372/659 (56.4)	210/457 (45.9)
Stratified according to age-group (years)		
⩽34	58/99 (58.6)	18/34 (52.9)
35–49	118/182 (64.8)	23/64 (35.9)
50–64	109/190 (57.4)	72/156 (46.2)
⩾65	87/188 (46.3)	97/203 (47.8)
		
*In situ* MM	131/220 (59.5)	49/127 (38.6)
Stratified according to age-group (years)		
⩽34	28/39 (71.8)	7/19 (36.8)
35–49	40/68 (58.8)	9/16 (56.3)
50–64	40/61 (65.6)	17/41 (41.5)
⩾65	23/52 (44.2)	16/51 (31.4)
		
Invasive MM	241/439 (54.9)	161/330 (48.8)
Stratified according to age-group (years)		
⩽34	30/60 (50.0)	11/15 (73.3)
35–49	78/114 (68.4)	14/48 (29.2)
50–64	69/129 (53.5)	55/115 (47.8)
⩾65	64/136 (47.1)	81/152 (53.3)

Abbreviations: MM=melanoma; SCREEN=Skin Cancer Research to provide Evidence for Effectiveness of Screening in Northern Germany.

**Table 3:** Age-standardised incidence rates (n/100 000 (95% CI)^a^; ASR (Europe)) of MM pre SCREEN, during the SCREEN project and post SCREEN, and absolute differences in incidence rates

	**SH**		**SL**		***P* for comparison of SH and SL**	
	**Women**	**Men**	**Women**	**Men**	**Women**	**Men**
*Incidence rate ASR (Europe)*						
Pre-SCREEN period (January 1998–December 2000)						
*In situ* MM (ICD-10 D03)	5.7 (5.0; 6.4)	3.7 (3.2; 4.3)	2.4 (1.8; 3.2)	1.0 (0.6; 1.6)	<0.001	<0.001
Invasive MM (ICD-10 C43)	16.8 (15.7; 18.0)	15.2 (14.1; 16.4)	9.2 (7.8; 10.6)	10.7 (9.3; 12.4)	<0.001	<0.001
SCREEN period (July 2003–June 2004)						
*In situ* MM (ICD-10 D03)	13.3 (11.5; 15.2)	7.7 (6.4; 9.2)	3.5 (2.1; 5.3)	3.1 (1.8; 4.8)	<0.001	<0.001
Invasive MM (ICD-10 C43)	25.7 (23.2; 28.3)	19.2 (17.2; 21.5)	10.9 (8.4; 13.8)	11.8 (9.2; 14.9)	<0.001	0.003
Post SCREEN and pre SCEDP period (January 2005–December 2007)						
*In situ* MM (ICD-10 D03)	10.4 (9.5; 11.4)	6.6 (5.9; 7.3)	4.0 (3.1; 5.0)	3.6 (2.8; 4.6)	<0.001	<0.001
Invasive MM (ICD-10 C43)	15.1 (14.0; 16.2)	15.1 (14.1; 16.3)	12.2 (10.6; 13.9)	11.5 (10.0; 13.1)	0.044	0.002
						
*Absolute differences in incidence rates ASR (Europe) (observed – preceding incidence as indicated above)*						
SCREEN period						
*In situ* MM (ICD-10 D03)	7.6 (5.6; 9.6)	4.0 (2.5; 5.5)	1.1 (−0.5; 2.7)	2.1 (0.6; 3.6)	<0.001	0.164
Invasive MM (ICD-10 C43)	8.9 (6.1; 11.7)	4.0 (1.6; 6.4)	1.7 (−1.3; 4.7)	1.1 (−2.0; 4.2)	0.005	0.373
Post SCREEN/pre SCS period						
*In situ* MM (ICD-10 D03)	−2.9 (−5.0; −0.8)	−1.1 (−2.7; 0.5)	0.5 (−1.2; 2.2)	0.5 (−1.2; 2.2)	0.019	0.264
Invasive MM (ICD-10 C43)	−10.6 (−13.3; −7.9)	−4.1 (−6.5; −1.7)	1.3 (−1.8; 4.4)	−0.3 (−3.4; 2.8)	<0.001	0.252

Abbreviations: ASR=age-standardised incidence rates; CI=confidence intervals; MM=melanoma; SCS=skin cancer screening; SCEDP=Skin Cancer Early Detection Program; SCREEN=Skin Cancer Research to provide Evidence for Effectiveness of Screening in Northern Germany; SH=Schleswig-Holstein; SL=Saarland.

a Poisson-based 95% CI.
